# Thermoplastic Starch–Based Composite Reinforced by Conductive Filler Networks: Physical Properties and Electrical Conductivity Changes during Cyclic Deformation

**DOI:** 10.3390/polym13213819

**Published:** 2021-11-04

**Authors:** Hamed Peidayesh, Katarína Mosnáčková, Zdenko Špitalský, Abolfazl Heydari, Alena Opálková Šišková, Ivan Chodák

**Affiliations:** 1 Polymer Institute of the Slovak Academy of Sciences, Dúbravská cesta 9, 845 41 Bratislava, Slovakia; hamed.peidayesh@savba.sk (H.P.); katarina.mosnackova@savba.sk (K.M.); zdeno.spitalsky@savba.sk (Z.Š.); abolfazl.heydari@savba.sk (A.H.); alena.siskova@savba.sk (A.O.Š.); 2 Institute of Materials and Machine Mechanics, Slovak Academy of Sciences, Dúbravská cesta 9, 845 13 Bratislava, Slovakia

**Keywords:** thermoplastic starch, conductive polymer composite, carbon black, electrical conductivity, mechanical deformation

## Abstract

Conductive polymer composites (CPC) from renewable resources exhibit many interesting characteristics due to their biodegradability and conductivity changes under mechanical, thermal, chemical, or electrical stress. This study is focused on investigating the physical properties of electroconductive thermoplastic starch (TPS)–based composites and changes in electroconductive paths during cyclic deformation. TPS–based composites filled with various carbon black (CB) contents were prepared through melt processing. The electrical conductivity and physicochemical properties of TPS–CB composites, including mechanical properties and rheological behavior, were evaluated. With increasing CB content, the tensile strength and Young’s modulus were found to increase substantially. We found a percolation threshold for the CB loading of approximately 5.5 wt% based on the rheology and electrical conductivity. To observe the changing structure of the conductive CB paths during cyclic deformation, both the electrical conductivity and mechanical properties were recorded in parallel using online measurements. Moreover, the instant electrical conductivity measured online during mechanical deformation of the materials was taken as the parameter indirectly describing the structure of the conductive CB network. The electrical conductivity was found to increase during five runs of repeated cyclic mechanical deformations to constant deformation below strain at break, indicating good recovery of conductive paths and their new formation.

## 1. Introduction

Conductive polymer composites (CPCs) are designed by mixing an insulating polymeric matrix with conductive fillers. Typically, conductive fillers are metallic powders or carbonaceous fillers containing graphite, carbon black, and carbon fibers [1]. Substantial effort has been devoted in the past decade to understanding the features of CPCs with respect to conductivity changes under external stimuli, such as thermal, electrical, mechanical, or chemical stresses [2]. Mechanical deformation is likely the most important factor that affects the structure of the conductive pathways. It can also lead to substantial destruction of the filler network structure. From this point of view, investigation of the dependence of electrical conductivity on mechanical deformation is of particular interest, especially if measured for electroconductive two–phase systems consisting of an insulating flexible polymeric matrix filled with an electroconductive filler. An electroconductive physical network is formed throughout the polymer matrix, enabling facile and rapid transport of electrons through the composite. It is well known that a sudden increase in conductivity occurs for these composites in a relatively narrow concentration range for the so–called percolation threshold of the filler [3]. Recently, polymers prepared from renewable resources as matrices for solid conducting composites, including starch [4,5,6,7,8,9,10,11,12], cellulose [13], pectin [14], and chitosan [15], have been investigated.

Starch has been considered as one of the most promising candidates in the biopolymer industry since it is abundant in nature, cost–effective, available from renewable resources, and biodegradable. However, to be used as a plastic material, it must be processable by standard plastic technologies. Thermoplastic starch (TPS) is a plasticized version of starch that can be obtained by the destruction of starch granules in the presence of plasticizers under heat and shear conditions [16,17,18]; however, the main shortcomings of TPS are its hydrophilic character, unsatisfactory mechanical properties, and poor thermal stability compared to conventional polymers [19,20,21].

Few studies have been carried out for CPCs based on TPS. In particular, Ma et al. [22] fabricated TPS membranes filled with multiwall carbon nanotubes by the casting method. TPS/carbon black (CB) membranes were also prepared by the microwave radiation method and melt extrusion [23]. Qian et al. used carbon black oxide particles as fillers in the TPS matrix to obtain well–dispersed particles in the mixture by the casting method and to increase the tensile strength of the composites [24]. Recently, electroconductive starch–based films plasticized by an ionic liquid have been investigated as a new preparation approach [25,26]. Furthermore, some other studies have demonstrated that fillers such as multiwalled carbon nanotubes, graphene, and graphene oxide can increase the tensile strength and improve the barrier and electrical properties of CPCs based on TPS [27,28,29].

In addition to studies comparing the electrical and mechanical properties of electroconducting CPCs, papers investigating both the mechanical properties and the electrical conductivity in parallel using online measurements are of particular interest. This approach was first suggested by Aneli et al. [30] and Flandin et al. [31] for detecting the changes in electroconductive pathways through vulcanized rubbers filled with conductive reinforcing carbon blacks. The method was elaborated in detail by us [32,33], enabling not only to describe the instant changes of the electroconducting (and reinforcing as well) pathways during mechanical deformation, but also to find clear correspondence of the changes in electrical conductivity of the composite filled with carbon blacks with features of stress–strain curves. In such a way, the decrease of conductivity during first stage of deformation, where, according to Hooke’s law, the deformation is proportional to strength and the slope represents Young’s modulus [34], is followed by an increase in conductivity after the change of the stress–strain curve from linearity to curving continuing by another conductivity decrease after passing the inflex point on the stress–strain curve. Moreover, similar behavior was also observed for thermoplastic polymers (e.g., polycaprolactone), indicating that the changes in reinforcing network of rubber at inflex point correspond to yield point of the thermoplastics [34]. In this regard, the electrical conductivity indirectly reveals the changes in filler structure related to mechanical deformation, indicating whether the conductive pathways are destroyed, remain intact, are reformed, or new electroconductive structures are formed, which should also lead to the extent of mechanical reinforcement of the material. The changes in electrical conductivity during mechanical deformation of elastomer–based composites, either in tensile or in compression mode, are described in the literature in a few cases [30,34]. However, to the best of our knowledge, TPS has not yet been investigated as a matrix in similar experiments. The objective of the present work is to determine rather delicate differences in the electroconductive behavior of TPS–CB composites. In all cases, the physical and mechanical properties, rheological properties, conductivity, and electrical behavior during deformation of the TPS–CB composites were evaluated. Furthermore, various effects were investigated, especially uniaxial deformation with varying strains, mechanical relaxation, and cyclic deformation.

## 2. Materials and Methods

### 2.1. Materials

Meritena^®^ 100 native corn starch was obtained from Brenntag (Bratislava, Slovakia). The water content was determined by drying in an oven at 105 °C for 5 h. Chezacarb A (Unipetrol RPA, Litvínov, Czech Republic) was used as a superconductive carbon black (CB). All of the data for this material were provided by the manufacturer and are summarized in Table 1. Glycerol was purchased from Centralchem, Ltd. (Bratislava, Slovakia). Double distilled water was used during the study.

### 2.2. Preparation of TPS–CB Composites

**Figure 1:**
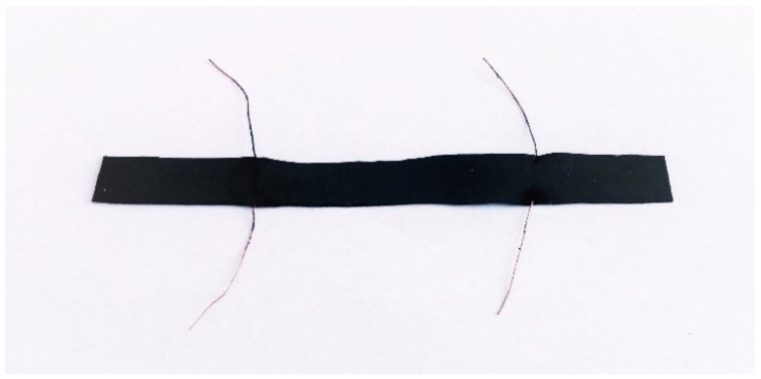
Specimen used for online measurements of electrical conductivity during mechanical deformation.

CB particles at 3, 10, 15, and 20 php (parts based on the dry weight of starch) corresponding to 1.7, 5.5, 8.1, and 10.5 wt%, respectively, were dispersed in a mixture of water and glycerol by applying sonication at ambient temperature for 1 h. The samples were marked as CB–X, where X is the CB amount in php. To obtain gelatinized starch, a suspension of starch, CB, glycerol, and water was heated at 80 °C for 10 min under continuous mechanical mixing. The weight ratio of starch/glycerol/water was 1.0:0.7:2.3. The obtained samples were dried at 100 °C for 5 h to remove excess water followed by holding at 60 °C overnight to prevent moisture absorption by the material. TPS–CB composites were then processed by kneading in a laboratory mixer Plastograph Brabender PLE 331 (Brabender GmbH, Viersen, Germany) for 10 min at 130 °C and 100 rpm. A 1-mm-thick slab was prepared by compression molding (laboratory press LabEcon 300, Fontijne Presses, Delft, The Netherlands) at 130 °C using 4 min of preheating without pressure and an additional 4 min at a pressure of 2.65 MPa. To easily remove the samples, two Teflon sheets were inserted between metal plaques and molds. To prepare the slabs for online measurements of the electrical current during deformation, two copper wires were inserted into the material during compression. Samples (0.7 mm thick) with dimensions of 100 × 10 mm were compression molded using a custom mold. Figure 1 shows the representative specimen used for online measurements.

### 2.3. Mechanical Testing

Dog–bone shaped specimens were obtained using pneumatic toggle press equipment. For each specimen, the dimensions of the deformed area during the tensile test were 3.5 × 30 mm, and the thickness of approximately 1 mm was precisely measured by using a digital caliper. The specimens were stored in airtight plastic containers for 24 h under ambient conditions prior to analysis, while the overall moisture content for each TPS sample was determined to be 5.7 ± 1.0%. An Instron 3365 universal testing machine (Instron, Norwood, MA, USA) was used to perform the tensile test. Tests were carried out at a speed of 1 mm/minute with a deformation of up to 1% and at a speed of 50 mm/min at higher deformations, according to ASTM D638. Seven replicates were prepared per formulation.

### 2.4. Online Measurements of Conductivity during Mechanical Deformation

The measurement of electrical current was performed in parallel with recording the stress–strain curve using a wireless TRMS multimeter (EXTECH Instruments, Shanghai, China). Mechanical testing was performed using the same machine as described in Section 2.3. The rates of deformation and voltage were 10 mm/min and 30 V, respectively. In this experiment, the electrical current was measured during five cycles of deformation with ultimate extensions of 2, 5, 10, and 20%. After the fifth cycle, performed up to preset deformation, the conductivity measurement was performed up to the break point.

### 2.5. Dynamic Mechanical Thermal Analysis (DMTA)

For DMTA measurements, a DMA Q800 (TA Instruments, Hüllhorst, Germany) analyzer was used in tensile mode at an amplitude of 20 μm and a frequency of 10 Hz. The TPS samples (ca. 10 × 7 × 1 mm^3^) were treated in the same way as for mechanical testing described in Section 2.3. The temperature range was fixed from −70 to 60 °C with a heating rate of 2 °C·min^−1^.

### 2.6. Electrical Conductivity

Before performing the measurements during deformation, the initial electrical conductivity of each material was measured using a Concept 40 (Novocontrol Technologies GmbH, Montabaur, Germany) for broadband dielectric spectroscopy. The measurement was performed using an instrument with an Alpha dielectric spectrometer provided by Novocontrol Technologies GmbH (Montabaur, Germany). Samples were prepared as disks with a diameter of 20 mm and a thickness of ~0.5 mm. All measurements were carried out in the frequency range of 0.1 Hz–1 MHz at room temperature. A BDS–1200 parallel–plate capacitor with two gold–plate electrodes was used as a test cell for the samples.

### 2.7. Rheological Measurements

The rheological properties of TPS–CB samples (circles with a diameter of 20 mm) were investigated using an AR 2000 rheometer (TA Instruments, Hüllhorst, Germany) equipped with a parallel plate geometry of 20 mm. The oscillatory mode measurements were conducted within the frequency range of 0.1–100 Hz. Before each measurement, the deformation amplitude was set up in the linear viscoelastic region determined by strain sweeps at a constant frequency.

## 3. Results and Discussion

The main concern of this work was aimed at the in situ characterization of the changes in electroconductive paths during mechanical deformation. The density and structure of these pathways was estimated according to the electrical conductivity; they can also contribute to the mechanical behavior of the materials under deformation and should result in changes in the rheological parameters and structure of the composite.

### 3.1. Electrical Conductivity

**Figure 2:**
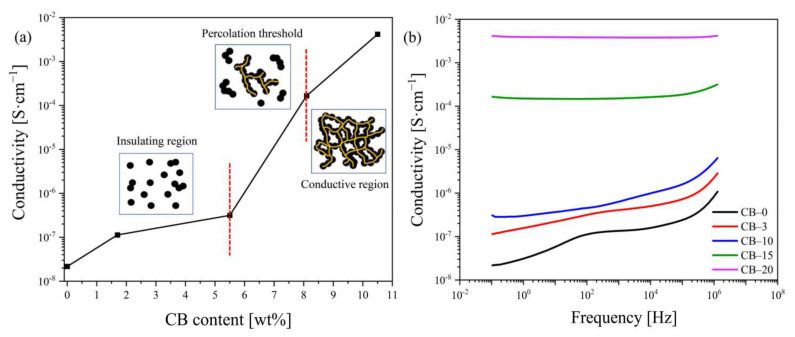
Electrical conductivity of TPS–CB composites as a function of (**a**) CB content and (**b**) frequency. The CB concentration is shown in php as the number after code CB–.

The electrical conductivity of TPS–CB composites measured under static conditions is plotted as a function of the filler content in Figure 2a. The conducting mechanisms are also schematically illustrated in Figure 2a, where the electrical conductivity of the TPS–CB composite changes from the insulating region to the conducting region. First, the electrical conductivity is increased by approximately one order of magnitude as a result of the addition of CB between 0 and 5.5 wt%. This fact indicates that the agglomerates of CB particles are still isolated, and conducting pathways are not formed in the TPS matrix (insulating region). A further increase in the CB concentration results in an increase in composite conductivity of over four orders of magnitude, from 3.1 × 10^−7^ to 4.2 × 10^−3^ S·cm^−1^ within the percolation concentration region between 5.5 and 10.5 wt% CB. In this region, the continuous conductive CB network is formed in the TPS matrix.

Figure 2b shows the frequency–dependent conductivities of TPS–CB composites for different filler contents. The electrical conductivity of TPS–CB composites increases with increasing CB content. Three different modes of dependence are observed for the TPS–CB composites (Figure 2b). For CB contents below the percolation threshold (i.e., 3 and 10 php), the composite exhibits typical behavior for an insulating polymer, where the conductivity increases almost linearly (in our case from ~10^−7^ to 10^−5^ S·cm^−1^) as the frequency increases from 1 Hz to 1 MHz. When the CB content lay approximately in the percolation threshold (CB–15), the second group showed constant electrical conductivity at low frequencies of 1–10^5^ Hz, whereas the conductivity increased at frequencies higher than 10^5^ Hz. Both isolated CB particles and conduction pathways exist in this composite. The conductivity increased with the frequency due to the growing importance of polarization effects [35]. The third mode of the course appears for the CB content above the percolation threshold (CB–20), where the absolute value of the conductivity remains constant with rising frequency, indicating the presence of stable conducting CB paths in the composite.

### 3.2. Mechanical Properties

Table 2 represents the mechanical data for the TPS–CB composites, including the ultimate tensile strength, elongation at break, and Young’s modulus values. It becomes apparent that the ultimate tensile strength of the TPS–CB composite increases with increasing CB content compared to that of neat TPS. On the other hand, the addition of CB to the TPS matrix leads to a decrease in elongation at break. The increase in the CB content from 0 to 10.5 wt% (corresponding to 0 to 20 php) results in a substantial enhancement in tensile strength from 0.30 to 3.18 MPa, while the Young’s modulus is increased from 0.81 to 32.77 MPa.

As expected, filling the TPS with rigid particles results in stiffer material due to the reinforcement effect of the CB particles. Reinforcement due to filler addition usually translates into a continuous decrease in elongation at break, concomitantly with an increase in Young’s modulus (rigidity), as matrix–filler interactions restrain chain mobility [23]. In other words, CB particles in the TPS matrix act as physical crosslinkers due to a certain degree of adhesion of matrix macromolecules onto the filler surface. Therefore, the increase in tensile strength and Young’s modulus of the TPS–CB composites are mainly related to CB particles affecting the polymer chain mobility during the elongation process.

**Figure 3:**
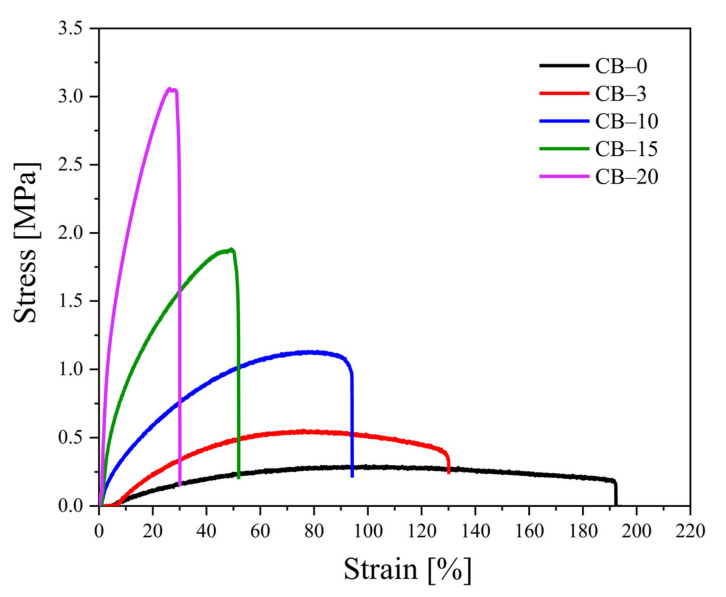
Stress–strain curves for TPS–CB composites containing various CB contents, in the picture the CB concentration is shown in php as the number after code CB–.

Figure 3 shows representative stress–strain curves for neat TPS and TPS–CB composites. A small linear elastic region followed by a nonlinear zone until the fracture point can be observed for all samples. Stress increases continuously with rising strain until fracture, which is typically referred to as plastic deformation. This behavior is similar to that indicated in the literature for starch–based films [36,37]. The addition of higher amounts of CB results in a progressive enhancement in maximum tensile strength along with a substantial decrease in elongation at break, indicating filler–matrix interactions.

### 3.3. Dynamic Mechanical Thermal Analysis (DMTA)

**Figure 4:**
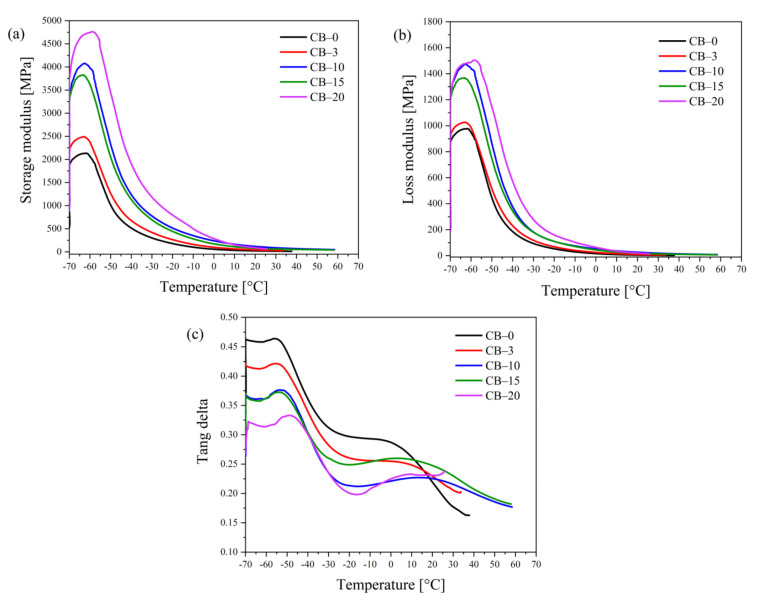
(**a**) Storage modulus, (**b**) loss modulus, and (**c**) tan δ curves for TPS–CB composites containing various CB contents. The CB concentration is shown in php as the number after code CB–.

The DMTA curves for the TPS–CB composites are shown in Figure 4. Figure 4a shows the storage modulus of the samples as a function of temperature. Below the glass transition, the storage modulus increases with increasing CB content in the composites, as expected. The increase in the storage modulus of the TPS–CB composite with a higher CB content should be attributed to the reinforcement effect of CB. Furthermore, the better dispersion of the CB particles in the TPS matrix leads to a substantial enhancement of the storage modulus of the TPS–CB composite. This explanation corresponds with the results from other published data for TPS composites filled with clay particles [38,39], including our previous publication [16].

The loss modulus and tan δ curves are presented in Figure 4b,c, showing a more complex evolution of the thermomechanical behavior in the presence of CB. The temperatures at which the maximum value for tan δ appears as determined from the data in Figure 4c are presented in Table 3. TPS typically shows two transition peaks in the DMTA curves. The alpha transition at low temperatures, T_α_, which is related to the low molecular weight plasticizer, and the beta transition, T_β_, which occurs at higher temperatures and belongs to the starch–rich domains described in the literature [40,41]. As depicted in Figure 4c, the addition of CB results in a temperature increase for the starch–rich phase transition. The shift in T_g_ to higher temperatures due to the incorporation of CB can be explained by the filler–polymer interaction and a lower degree of chain freedom [42]. The interfacial interaction between the TPS matrix and fillers is an essential factor affecting the starch backbone stiffness. CB particles act as a physical crosslinker of starch molecules, thus increasing the glassy state transition temperature of the starch–rich phase. However, the agglomeration of CB particles can lead to an opposite effect due to the decrease in the values for the effective filler–polymer interaction and the number of physical crosslinking points. Certainly, this effect is expected to manifest itself mainly at higher CB concentrations, which is supported by the changes in the transition temperatures shown in Table 3. The lower transition temperature corresponding to T_g_ is almost identical for all composites with CB contents up to 15 php, while the material containing 20 php exhibits a T_g_ that is 10 °C higher. On the other hand, the second peak appears at the highest temperature for TPS containing 10 php CB, which is the CB concentration at the beginning of the percolation area, while the excess CB results in certain softening of the material due to increased CB agglomeration, which is assumed to contribute to lower effective contact for the CB surface with starch.

### 3.4. Rheological Measurements

**Figure 5:**
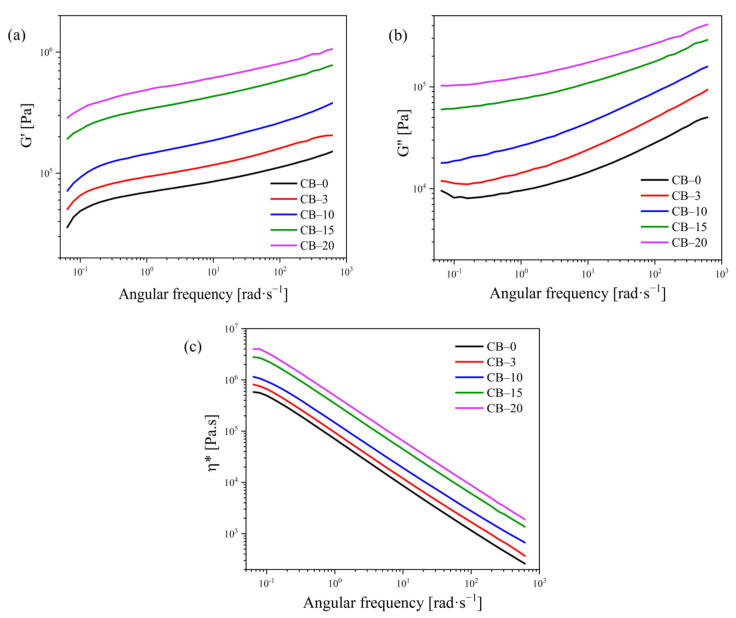
Dependence of (**a**) storage modulus *G*′ and (**b**) loss modulus *G*″ vs. frequency for neat TPS and after addition of 1.7, 5.5, 8.1, and 10.5 wt% CB loading (corresponding to 3, 10, 15, and 20 php in sample codes, respectively).

Electroconductive polymer composites containing conductive macrofillers are generally highly filled materials to provide sufficient electrical conductivity [43]. High filler content can dramatically affect the viscoelastic properties of the polymer matrix and worsen processability. Therefore, rheological measurements in the melt are important tools for understanding the effects of filler type and its concentration on material behavior since the data may indicate possible structural changes such as crosslinking, degradation, and chain recombination [44]. To study the structural variations, the linear viscoelastic behavior in the melt of TPS samples filled with various amounts of CB was investigated by oscillatory shear measurements at 150 °C. Figure 5 shows frequency sweeps as dependences of (a) storage modulus *G′*, (b) loss modulus *G″*, and (c) complex viscosity (η*) on the angular frequency (ω) for neat TPS and TPS–CB composites with varying filler loading ranging from 1.7 wt% to 10.5 wt%. It is clearly observed that the increase in shear moduli/η* linearly depends on increased filler loading as a result of an effective reinforcement effect of the CB. Moreover, the slope of the *G′* curves in the low–frequency region slightly decrease with increasing filler content and become less frequency–dependent (the increment of *G′* decreases with increasing ω). This phenomenon is well known, especially for nanocomposites exhibiting “gel–like” (solid–like) behavior, and is attributed to the formation of a physical network by fillers. To determine the gel–like behavior, the dependence of *G′* at the lowest frequency was used as a function of CB loading, similar to Kasgoz [43]. These authors reported that the increment in *G′* at ω of 0.1 rad·s^−1^ depends on the filler content and increases dramatically above a critical volume of the filler fraction defined as the rheological percolation threshold (*φ*_p_). Generally, the *φ*_p_ values are related to the formation of fractal flocs within composite systems, and rheological percolation, as such, does not require physical contact between filler particles and is more likely due to the hydrodynamic effects of fillers on the polymer melts. Kotsilkova et al. [45,46] pointed to the existence of two critical concentrations describing the first and second percolation thresholds. While the first *φ*_f_ represents only local percolation based on fractal floc formation, the second *φ*_p_* is directly related to the formation of a continuous three–dimensional floc network. Zhu et al. [47] also used a low amplitude oscillatory shear test for the determination of two percolation thresholds by comparison of *G′* and *G″* values at a ω of 1 rad·s^−1^.

**Figure 6:**
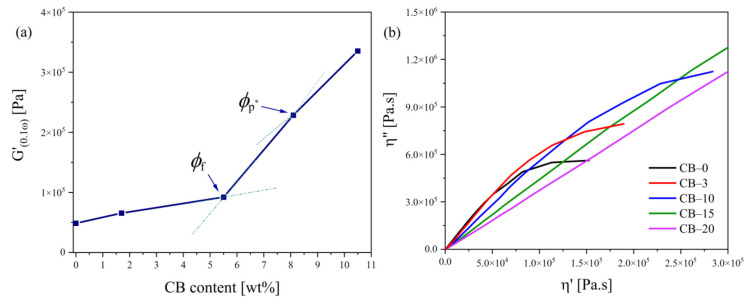
(**a**) Dependence of storage moduli for TPS–CB composites at an angular frequency of 0.1 rad·s^−1^ (*G′*_(0.1ω)_) as a function of volume fraction of CB for estimating the critical volume fraction (*φ*_p_) and (**b**) Cole–Cole plots at 150 °C for neat TPS and TPS–CB composites filled with various concentrations of CB (1.7, 5.5, 8.1, and 10.5 wt% of CB loading corresponding to 3, 10, 15, and 20 php in sample codes, respectively).

As shown in Figure 6a, the *φ*_f_ and *φ*_p*_ values for TPS filled with CB were found to be 5.5 wt% and 8.1 wt%, which correspond to CB–10 and CB–15, respectively, and are almost identical to the percolation threshold region valid for electrical conductivity. The first critical concentration *φ*_f_ was determined from the crossing point of the first two distinctive slopes. Zhu et al. [47] used the crossing point of *G′* and *G″* curves to determine the second percolation threshold. In our case, the TPS–CB composites showed gel–like behavior in the full frequency range, and the *G′*–*G″* crossing point could not be evaluated in this manner. Therefore, the second percolation threshold was determined in a similar way to the Kasgoz et al. approach [43].

Figure 6b shows the Cole–Cole plots for neat TPS (*η″* vs. *η′*) and its composites with various CB loadings. The presence of different concentrations of filler remarkably affects the rheological properties of TPS. At a lower CB loading of up to 5.5 wt%, viscosity curves are arc–shaped similar to that for unfilled TPS but show a tendency towards linearity through a straightening of the arc, especially with increasing filler content. On the other hand, courses with completely different shapes are observed for composites filled with higher CB amounts, reaching linear variation. The linear variation of the Cole–Cole plots is frequently associated with the densification of a 3D network. Many reports [44,48,49,50] show an increase in the slopes of linear Cole–Cole plots, which are usually due to either invasive treatments such as irradiation leading to (chemical) crosslinking within the polymer or the addition of an interaction–capable compatibilizer/filler increasing polymer–filler interactions. Thus, the change in the Cole–Cole plot indicates that a critical filler concentration above the stable 3D reinforcing physical network of filler particles is formed [49].

### 3.5. Conductivity during Uniaxial Deformation

**Figure 7:**
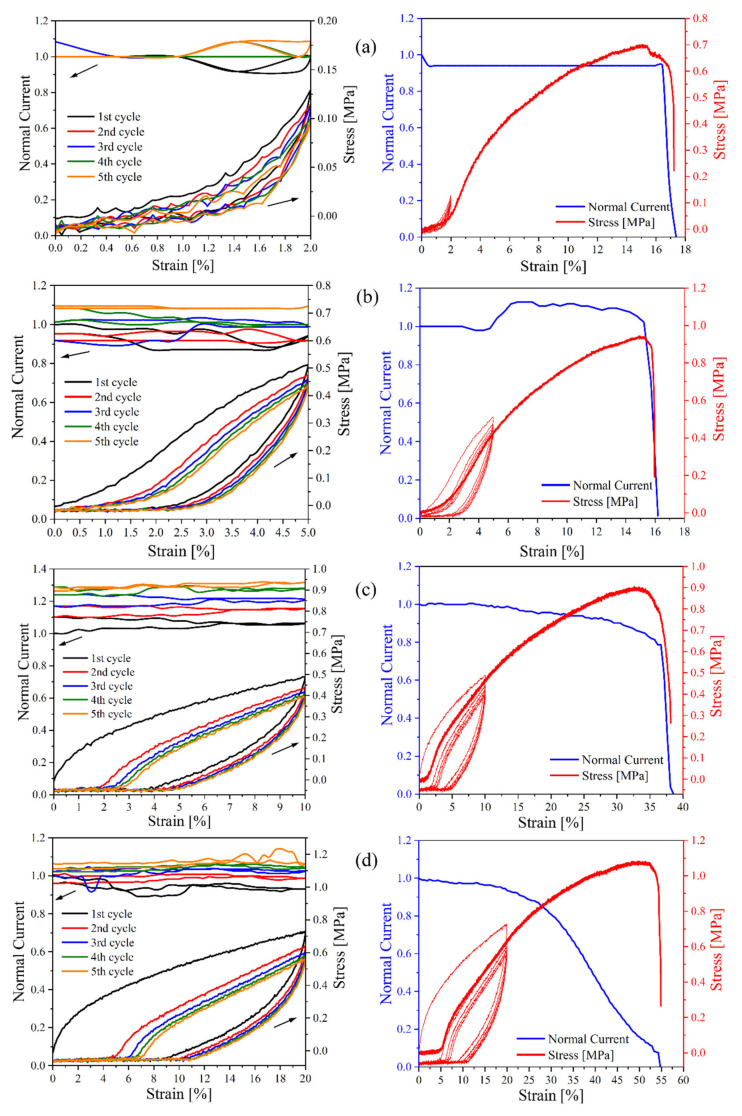
Dependence of normalized current and stress on deformation during five runs of cyclic deformation (left–hand side) and up–to–break deformation (right–hand side) for CB–15, containing 15 php CB, after various cyclic strains of (**a**) 2%, (**b**) 5%, (**c**) 10%, and (**d**) 20%.

**Figure 8:**
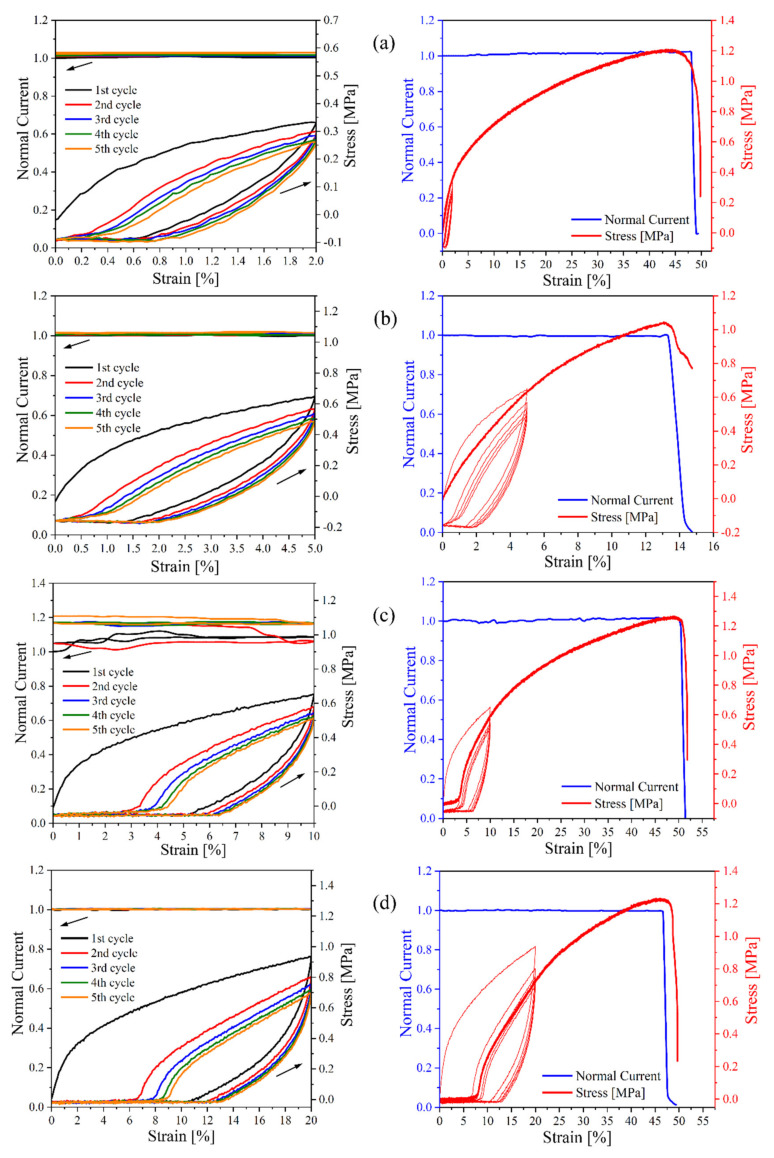
Dependence of normalized current and stress on deformation during five runs of cyclic deformation (left–hand side) and up–to–break deformation (right–hand side) for CB–20, containing 20 php CB, after various cyclic strains of (**a**) 2%, (**b**) 5%, (**c**) 10%, and (**d**) 20%.

Since parallel measurements of stress–strain curves and electrical conductivities require the material to be studied to have a rather high conductivity, only composites with a CB content above the percolation threshold could be used. Therefore, only two materials were tested, namely, CB–15 and CB–20, during five runs of cyclic deformations with various ultimate extensions of 2, 5, 10, and 20%, followed by deformation up to the break point after each set of cyclic runs. The courses are presented in Figure 7 and Figure 8, respectively. The current slightly increases during each consecutive deformation cycle, while all dependencies are almost identical in the rising–strain period. It is assumed that higher conductivity during consecutive deformation cycles indicates the formation of new conductive paths or healing of minor defects in current paths, resulting in good conductivity recovery during deformation. Table 4 represents the absolute values for the current at the beginning of the first cycle and the up–to–break run. The conductivity at the starting point of the up–to–break experiment is higher than that at the beginning of the first cycle. All these findings indicate the existence of good conductivity path recovery after cyclic deformation. Mechanical deformation appears to be the most crucial factor affecting the structure of the conductive pathways [51]. Generally, it leads to substantial destruction of the filler network structure, but the massive formation of new paths has also been reported, especially in rubber [52] and thermoplastics, e.g., polycaprolactone [30,34,53]. For the composite with a starch matrix containing a CB content well above the percolation concentration, an almost constant current is observed for all cycles, as illustrated in Figure 8. This implies that in TPS creates much lower shear stress during mechanical deformation; the forces generated are not sufficient to destroy the conductive CB paths, especially since deformations are much smaller than those reported for rubber.

For the CB–15 composite (Figure 7), a decrease in conductivity before the break is observed due to the destruction of the conductive pathways formed by the filler [34]; however, the conductivity of CB–20 is interestingly constant before it breaks, as shown in Figure 8. This means that the number of conductive pathways is higher in CB–20. Herein, this confirms that a core of the conductive filler structure is sufficiently strong to maintain constant conductivity and remain intact. However, mechanical deformation might result in the decay of some pathways.

The stress–strain cycles exhibit standard shapes with typical hysteresis features, where during increasing deformation, the stress is significantly higher than the values measured at the same strain but during a decrease in deformation. It is worth noting that the viscoelasticity manifesting itself in the TPS results in generating a hysteresis loop, which means that a large amount of mechanical energy is dissipated during the first extension cycle [54]. A decrease in loading and unloading stress occurs in the cycles because of the plastic deformation and hysteresis effect of the composite with the thermoplastic matrix [55]. On the other hand, evident signs of permanent deformation are observed for the composites, especially for cyclic deformations of 10% and higher. However, even in this case, permanent deformation exhibits itself from comparison of only the first and second runs, while the permanent deformation is almost nonexistent for the following cycles.

## 4. Conclusions

TPS–CB composites were obtained in the present study by incorporating CB conductive fillers into the plasticized starch matrix. To achieve well–dispersed CB particles in the TPS matrix, a combination of both solvent dissolution and melt processing was performed by ultrasonication and the use of a Plastograph Brabender mixer, respectively. The correlations between the TPS–CB composite percolation thresholds and morphologies were confirmed by conductivity measurements. The reinforcing effect of CB particles results in a remarkable increase in tensile strength and Young’s modulus but a substantial decrease in deformation at break. The electrical conductivities of the TPS–CB composites increase with increasing CB loading. According to the percolation theory, TPS–CB composites form a three–dimensional conductive network at CB concentrations over 5.5 wt%, which is defined as the percolation threshold for the system investigated. Furthermore, the electrical conductivity increases or at least remains constant during five runs of repeated cyclic mechanical deformations to constant deformation below strain at break, indicating more perfect recovery of conductive paths and their new formation. This work provides new insight for designing and constructing conductive materials based on starch, which are promising for electromagnetic shielding applications.

## Figures and Tables

**Table 1:** Properties of the carbon black (CB) used as filler.

Property	Amount
Iodine adsorption	900−1200 mg/g
DBF adsorption	340−420 mL/100g
Specific area	800 m^2^/g
Particle diameter	2−20 nm

**Table 2:** Mechanical properties, including tensile strength, elongation at break, and Young’s modulus of the TPS–carbon black (CB) composites.

Sample Code	CB Content (wt%)	Tensile Strength (MPa)	Elongation at Break (%)	Young’s Modulus (MPa)
CB–0	0	0.30 ± 0.02	193.55 ± 25.21	0.81 ± 0.19
CB–3	1.7	0.56 ± 0.01	130.23 ± 13.51	2.26 ± 0.17
CB–10	5.5	1.12 ± 0.06	94.05 ± 5.88	5.60 ± 0.50
CB–15	8.1	1.81 ± 0.26	51.76 ± 17.84	16.24 ± 2.97
CB–20	10.5	3.18 ± 0.16	29.88 ± 8.12	32.77 ± 4.51

**Table 3:** Temperatures at which maximum values for tan δ appear as extracted from Figure 4c.

Sample Code	1st Peak T (°C)	2nd Peak T (°C)
CB–0	−56.1	3.3
CB–3	−55.5	9.9
CB–10	−53.2	12.8
CB–15	−54.2	3.2
CB–20	−48.8	9.3

**Table 4:** The values of the current measured at the starting point of cyclic deformation and up–to–break experiments.

Sample Code	Starting Point of	Current (μA)			
		2% Strain	5% Strain	10% Strain	20% Strain
CB–15	Cyclic deformation	1.18	1.05	1.29	1.34
	Up–to–break	1.23	1.14	1.83	1.79
CB–20	Cyclic deformation	2.44	2.68	2.45	3.64
	Up–to–break	2.54	2.76	2.94	3.67
